# Factors influencing the COVID-19 vaccine uptake among Ukrainian refugees in Poland: a cross-sectional study

**DOI:** 10.3389/fpubh.2026.1786387

**Published:** 2026-04-30

**Authors:** Oskar Pasek, Łukasz Duda-Duma, Klaudia Pyzio, Daria Lizurej, Anna Sikora, Maria Ganczak

**Affiliations:** 1 Department of Infectious Diseases, Collegium Medicum University of Zielona Góra, Zielona Góra, Poland; 2 University Hospital in Zielona Góra, Zielona Góra, Poland; 3 Provincial Hospital in Kędzierzyn-Koźle, Kędzierzyn-Koźle, Poland; 4 Student Research Group, Collegium Medicum, University of Zielona Góra, Zielona Góra, Poland

**Keywords:** COVID-19, COVID-19 vaccination, COVID-19 vaccine uptake, Ukrainian migrants, Ukrainian refugees

## Abstract

**Background:**

The COVID-19 vaccination of Ukrainian refugees (UR) should be a top priority.

**Methods:**

Between May and July 2023, we conducted a cross-sectional study that surveyed consecutive UR registering at a refugee center in Zielona Gora, Poland, about COVID-19 vaccination and influencing factors. A self-administered, anonymous questionnaire was used.

**Results:**

Response rate was 83.4%. A total of 837 Ukrainians participated in the study, including 41.6% males. The largest age group (31.6%) was between 31 and 40 years old. Most respondents (61.2%) had previously lived in Ukrainian cities with fewer than 250,000 inhabitants. The majority were single (68.9%), and 57.5% reported low socioeconomic status (SES). Slightly over half of the participants (51%) had graduated from primary or vocational school. Less than one-fifth of respondents (18.3%) had completed the full COVID-19 vaccination course. The most frequently reported motivation for vaccination was protection of one’s own health (27.1%), followed by protection of family members’ health (13.3%). In addition, 27.8% reported receiving an influenza vaccination within the last 5 years, 22.8% had previously been infected with SARS-CoV-2, and 25.6% reported COVID-19 infection in a family member. The multilevel logistic regression model showed strong associations between COVID-19 vaccination and prior SARS-CoV-2 infection (OR = 7.39; *p* < 0.0001), regular influenza vaccination (OR = 3.34; *p* < 0.0001), origin from a Ukrainian city with more than 250,000 inhabitants (OR = 1.89; *p* = 0.0001), and willingness to travel abroad (OR = 1.89; *p* = 0.0001).

**Conclusion:**

The uptake of the full COVID-19 vaccination course among UR in Poland was disturbingly low. UR seek vaccination primarily for altruistic motives, i.e., to protect themselves and others. Educational programs should emphasize the benefits of the COVID-19 vaccine and raise awareness of its importance, effectiveness, and safety, with targeted outreach to high-risk groups identified in this study.

## 1 Introduction

The SARS-CoV-2 pandemic, which causes COVID-19 disease, is one of the most significant threats to have affected humanity in the last century. This remained a major health challenge in 2022–2023 (1). As the pandemic’s impact declines with rising vaccination rates, improvements are expected (2). Recent studies have demonstrated that COVID-19 vaccines improve protection against severe disease and symptomatic infection and reduce hospitalization (3). Moreover, they minimize mortality by enhancing both humoral and cellular anti-SARS-CoV-2 immunity, particularly in vulnerable populations. A study based on data from the Israeli Ministry of Health shows that in the general population, the risk of infection decreased by 88–92% after the third dose of the COVID-19 vaccine. Additionally, among immunocompromised patients, the seroconversion rate increased from 39 to 66% (4).

The Russian invasion of Ukraine has contributed to one of the largest migrations in Europe recently. Since February 24, 2022, Poland has been primarily affected by the arrival of Ukrainian citizens due to the ongoing armed conflict. As of January 31, 2024, approximately 18.8 million Ukrainian refugees had entered Poland (5); as of May 31, 2024, 1,477,759 Ukrainians held valid residence permits in Poland (5). These numbers clearly illustrate the scale and nature of this crisis, which continues to pose a significant humanitarian and public health challenge for Poland. Therefore, the COVID-19 vaccination of UR should be a top priority. This may be more feasible than delivering immunization programs in-country (6).

Vaccine hesitancy and refusal are significant in many societies. Hence, the World Health Organization (WHO) recently identified them as among the top 10 threats to global health (7). Before the war, COVID-19 vaccination rates in Ukraine were among the lowest in Europe (8). By February 2022, less than 40% of the Ukrainian population had received one dose of the COVID-19 vaccine; 35% had completed the full course, compared with an average of 65% in the rest of Europe (9). Additionally, it is suspected that some vaccination certificates issued to Ukrainians are fraudulent (10).

According to the literature, the most prominent factors influencing acceptance of the COVID-19 vaccine are age, gender, education, income level, and race/ethnicity. These also include self-protection against COVID-19 (11). Some intrapersonal factors, such as personal preferences, concerns about COVID-19 vaccines, history and perception of vaccinations in general, knowledge about COVID-19 and health, also have a strong influence (12).

High COVID-19 vaccination coverage is essential, specifically among vulnerable communities, such as Ukrainian refugees (UR), as they may be the source of infection for the entire population (13). Insights into the barriers and drivers of URs’ COVID-19 vaccination uptake can inform the development of effective policies and interventions to promote positive vaccination behaviors (14). Hence, we conducted a study on vaccination uptake and its determinants among UR living in Poland.

## 2 Methods

### 2.1 Study design

A cross-sectional survey was conducted between 5 July and 24 August 2023.

### 2.2 Study population

Consecutive Ukrainian refugees visiting the Department of Civil Affairs at Zielona Góra City Hall, Poland were invited to participate in the study. The Department assists foreign nationals with the legalization of their stay. Although the service was provided to individuals of various nationalities, we selected Ukrainian citizens as the study population. They were informed that the survey was anonymous, participation was voluntary, and they could withdraw from the study at any time.

### 2.3 Data collection

Six medical students, members of the Student Research Group, along with two PhD students from the Department of Infectious Diseases at the University of Zielona Góra, Poland, distributed questionnaires among the UR. An anonymous paper questionnaire, developed by the research team following a thorough literature review (11, 12, 15–18), was used as the data-collection tool. Respondents completed it within 10–15 min. The questionnaire comprised 44 items, organized into several sections covering demographics (age range, gender, marital status, education, socioeconomic status (SES), place of residence in Ukraine); personal experience with influenza; COVID-19 vaccination status (number of doses received), reasons for taking the third dose of the COVID-19 vaccine; and vaccine-related attitudes.

Attitudes were assessed by giving 1 point for each correct answer (a minimum of 0, a maximum of 5 points). Questions were divided into statements regarding self-perceived immunity to COVID-19, awareness of the effectiveness of SARS-CoV-2 vaccination, the appropriateness of vaccination before travel, and potential benefits of vaccination.

The representative target sample size was calculated with a sample size calculator (19). This resulted in 664 participants (the minimum recommended survey size), using a margin of error of ±5%,

a confidence level of 99%, and a 50% response distribution. Variables included in the logistic regression model were selected based on univariate analysis (*p* < 0.05). The questionnaire was intended for adult participants only (≥18 years old). Minors were not allowed to participate in the study. Exclusion criteria included incorrectly completed questionnaires, such as selecting more than one answer when only one was allowed (e.g., marking both “yes” and “no”), missing responses to required questions, or marking answers in an unclear or ambiguous way that made verification impossible. These questionnaires were excluded from the final analysis.

### 2.4 Ethics statement

Written confirmation from the Bioethics Committee of the University of Zielona Góra (RCM-CM-KBUZ.0015.13.2024) was obtained before conducting this research.

### 2.5 Statistical analysis

Categorical variables were presented as frequencies and percentages, and continuous variables were reported as means. COVID-19 vaccine uptake was a primary outcome variable. Demographic characteristics were assessed using bivariate analysis as follows: age (in years), gender, residence in Ukraine (city with ≤250,000 or >250,000 residents), and marital status (single/in relationship), education level (higher/lower education), SES (good and very good/low and medium), influenza and COVID-19 vaccination—ever (yes/now/I do not know). The Shapiro–Wilk test indicated that the variables were not normally distributed (*p* < 0.005). So, the non-parametric tests were used to assess the significance of the difference. The χ^2^-test was used for 2-group comparisons of categorical variables. For numerical variables, the Mann–Whitney test was used. For contingency table cells with expected counts less than 5, the Yates correction was applied. The size of the effect was assessed using the Cramer *φ* coefficient.

Logistic regression models were developed to analyze factors associated with the likelihood of receiving 1 or 2 doses of the COVID-19 vaccine. Final associations between predictors and outcomes, adjusted for covariates, were assessed using logistic regression coefficients. Regression results are presented as odds ratios (ORs) together with 95% CI for individual predictors. As a measure of the model’s fit to the data, the Pseudo R^2^ Nagelkerke (R^2^N) was adopted, which allows assessment of the proportion of variance explained by the model (0.3–0.5: average fit). A *p* value was statistically significant if <0.05. All statistical analyses were carried out using the Statistica 13.3 package (StatSoft Polska, 2017).

## 3 Results

### 3.1 Demographics

A total of 950 questionnaires were distributed, and 837 responses were received, yielding a response rate of 88.1%. Women accounted for 54.8% of respondents (Table 1). The age distribution was as follows: ≤20 years, 9.8%; 21–30 years, 26.5%; 31–40 years, 33.1%; 41–50 years, 18.4%; 51–60 years, 8.8%; >60 years, 3.4%. Among respondents, 61.2% were in a relationship, and the remainder were single. The majority of respondents (57.0%) reported low or medium SES. Basic or secondary education accounted for 50.5%, whereas the remainder pursued vocational or higher education. A total of 63.6% had lived in Ukrainian cities with populations ≤250,000.

**Table 1:** Demographic characteristics of the studied population; Zielona Gora, Poland, 2023 (*n* = 837).

Variable	Class	*N*	%
1. Gender	Female	489	54.8
	Male	352	42.1
	Other	26	3.1
2. Age	≤40 years	581	69.4
	>40 years	256	30.6
3. Marital status	Single	158	18.9
	In relationship	679	81.1
4. Education	Primary/vocational	414	49.5
	High school/university degree	423	50.5
5. Residence in Ukraine	≤250,000	532	63.6
	>250,000	305	36.4
7. How long have you lived in Poland	≤6 months	378	45.2
	>6 months	459	54.8
8. How do you assess your financial status?	Poor/medium	477	57.0
	Good/very good	360	43.0
9. Has a family member contracted COVID-19?	No/I do not know	623	74.4
	Yes	214	25.6
10. Have you been vaccinated against influenza in the last 5 years?	Yes	233	27.8
	No/I do not remember	604	72.2
11. Have you ever been infected with SARS-CoV-2?	Yes	191	22.8
	No/I do not know	646	77.2
12. Have you ever received COVID-19 vaccine?	No	507	60.6
	Yes	330	39.4

### 3.2 COVID-19 vaccine uptake

**Figure 1:**
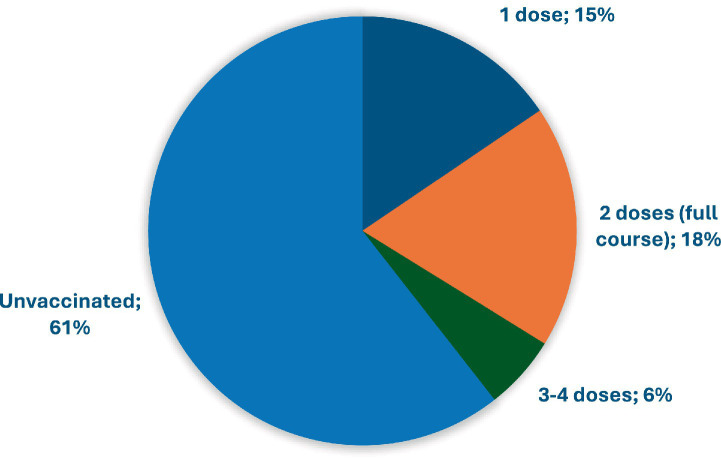
COVID-19 vaccine uptake by the number of doses; Zielona Gora, Poland, 2023 (*n* = 837).

The study found that 15.5% (*n* = 130) of UR received only the first dose of the COVID-19 vaccine, 18.3% (*n* = 153) received the full course, and about 5.6% (*n* = 47) three or four doses: the rest (60.6%, *n* = 507) were unvaccinated (Figure 1).

UR were also asked which COVID-19 vaccine they had received as their first dose. A quarter of the respondents (25.2%) indicated the Pfizer vaccine, 17.6% Moderna, 17.0% Johnson and Johnson, and 12.7% AstraZeneca. The other subjects reported receiving the Sinovac/Covishield vaccine or did not recall which vaccine they received as the first dose.

### 3.3 Vaccination with at least one dose and its determinants

Table 2 presents factors related to COVID-19 vaccination (any dose). A statistically significant association was observed between the size of the city of origin in Ukraine, as well as between the economic situation and the frequency of COVID-19 vaccination, with similar effects (*p* < 0.05): 36.65% of people residing in Ukrainian cities with a population of up to 250,000 and 44.26% of people from larger cities declared vaccination against COVID-19; *p* < 0.03. Respondents with an average or worse economic situation accounted for 36.48% of those vaccinated, while 43.33% in at least good condition declared vaccination; *p* < 0.04.

**Table 2:** COVID-19 vaccination by selected demographic variables; Zielona Gora, Poland, 2023 (*n* = 837).

Variable	COVID-19 vaccination		*N*	*p*
	No	Yes		
Gender				
Female	273	186	459	
%	59.5%	40.5%		
Male	220	132	352	
%	62.5%	37.5%		
Age group				0.44
≤40	357	224	581	
%	61.5%	38.6%		
>40	150	106	256	
%	58.6%	41.4%		
Marital status				0.62
In relationship	414	265	679	
%	61.0%	39.0%		
Single	93	65	158	
%	58.9%	41.1%		
Education				0.86
Primary/vocational	252	162	414	
%	60.9%	39.1%		
Medium/higher	255	168	423	
%	60.3%	39.7%		
City of origin in Ukraine				**0.03**
≤250,000	337	195	532	
%	63.6%	**36.7%**		
>250,000	170	135	305	
%	55.7%	**44.3%**		
Duration of stay in Poland				0.09
≤6 months	241	137	378	
%	63.8%	36.2%		
>6 months	266	193	459	
%	58.0%	42.1%		
**SES**				**0.04**
Poor/medium	303	174	477	
%	63.5%	**36.5%**		
Good/very good	204	156	360	
%	56.7%	**43.3%**		

Bold values indicates statistically significant results.

Among UR who had been infected with SARS-CoV-2, more than 80% were vaccinated against

COVID-19, whereas in a group who had not experienced infection, only 27.40% were vaccinated (*p* < 0.0001); Table 3. Respondents, who have been vaccinated against influenza in the last 5 years, were more likely to be immunized against COVID-19 (68.24% vs. 28.31%; *p* < 0.0001). Factors related to personal experience with the disease (|*φ*| = 0.45) and flu vaccination habit (|φ| = 0.37) had the most significant impact on COVID-19 vaccination. Slightly smaller, but the experience of the disease among family members also had a considerable effect (|φ| = 0.22).

**Table 3:** COVID-19 vaccination by selected variables; Zielona Gora, Poland, 2023 (*n* = 837).

Variable	COVID-19 vaccination		*N*	*p*
	No	Yes		
SARS-CoV-2 infection ever				**<0.0001**
Yes	38	153	191	
%	19.9%	**80.1%**		
No/I do not remember	469	177	646	
%	72.6%	**27.4%**		
Vaccination against flu in the last 5 years				**<0.0001**
Yes	74	159	233	
%	31.8%	**68.2%**		
No/I do not remember	433	171	604	
%	71.7%	**28.3%**		
COVID-19 (illness or death) of a family member				**<0.0001**
Yes	91	123	214	
%	42.5%	**57.5%**		
No/I do not remember	416	207		
%	66.8%	**33.2%**		

Bold values indicates statistically significant results.

### 3.4 Main determinants of vaccination against COVID-19 with the next dose

Eight hundred four respondents answered the question regarding the primary reason for the next dose of the COVID-19 vaccine. In total, respondents indicated 1,340 responses (Table 4). The motivation to protect one’s own health was most often indicated (27.1% of responses; 45.2% of respondents).

**Table 4:** Reasons for taking the next dose of the COVID-19 vaccine, Zielona Gora, Poland (*N* = 804).

Variable	*N*	% answers*	% cases*
Protecting health	363	27.1	45.2
Protecting my family’s health	178	13.3	22.1
I do not know	137	10.2	17.0
Work or/school requirements	119	8.9	14.8
Friend recommendation	116	8.7	14.4
Protecting the health of my community	96	7.2	11.9
Protecting the health of my colleagues	86	6.4	10.7
Travel abroad	85	6.3	10.6
Return to social life	68	5.1	8.5
I would not take the next dose of the COVID-19 vaccine	53	4.0	6.6
Government recommendations	39	2.9	4.9
*N*	1,340	100.0	–

*The column “Answers” refers to the total number of selected responses; the column “Cases” indicates the number and percentage of respondents who selected a given option.

### 3.5 Logistic regression model

The logistic regression model (R^2^L = 0.75) examined the association between vaccination with at least one dose of the COVID-19 vaccine and several factors, including sociodemographic characteristics, prior SARS-CoV-2 infection, and vaccination motivations (Table 5). The model showed strong relationships between vaccination and the following factors: experience of SARS-CoV-2 infection (OR = 7.39; *p* < 0.0001), flu vaccination habit in the last 5 years (OR = 3.34; *p* < 0.0001), origin from a city in Ukraine with more than 250,000 inhabitants (OR = 1.89; *p* = 0.0001), and travel (OR = 1.89; *p* = 0.0001).

**Table 5:** Multivariable logistic regression analysis: factors associated with the chances of being vaccinated against COVID-19.

Factors that increase the chances of being vaccinated against COVID-19*	OR	95% CI	95% CI	*p*
SARS-CoV-2 infection	7.39	4.84	11.32	<0.0001
Willingness to travel abroad	1.85	1.11	3.10	0.02
City of origin in Ukraine (>250,000)	1.89	1.35	2.70	0.0002
Flu vaccination	3.34	2.29	4.91	<0.0001

*R^2^N (Pseudo R^2^) = 0.32.

## 4 Discussion

### 4.1 Results overview

Sixty-one percent of UM in Poland were not vaccinated against COVID-19. A multivariate logistic regression analysis revealed that a history of COVID-19 infection was significantly associated with higher vaccine uptake (any dose). Vaccination against influenza in the past 5 years, living in large cities in Ukraine, and willingness to travel abroad were also significant predictors of vaccine uptake. The primary reasons for the next immunization dose are to protect one’s health and/or to protect the health of family members.

### 4.2 Vaccination rates among Ukrainian migrants

In this study, fewer than 1 in 5 UR received the full course of the COVID-19 vaccine. The low vaccination rates may be due to a long history of vaccine hesitancy in Ukraine, dating back to Soviet times, when vaccination was poorly enforced. The country is currently witnessing a historically low level of trust in the health system and authorities (20). According to a Wellcome Trust survey (2019), only 29% of Ukrainians believed vaccines were safe, while 50% thought they were effective (21). In addition, according to some Ukrainian authorities, a targeted misinformation campaign by Russia may also play an important role in vaccine hesitancy (22, 23). The military conflict in Ukraine provided favorable conditions for the dissemination of such false information (23).

Dramatically low COVID-19 vaccination rates among the UR were observed in this study, not exceeding the officially reported rate for Ukraine (38.4%). This could reflect the discrepancy between official reports and the actual number of vaccinations administered to citizens. One possible reason could be vaccine forgery (24). Notably, previous studies have shown that bribery inherited from the socialist period persists in Ukraine (25), including a black market in counterfeit COVID-19 certificates (10, 22, 26). Vaccine forgery typically involves a network of physicians who enter false vaccination records into medical registries. The lack of responsibility for such corruption creates conditions for further falsification of official documents.

Almost three-quarters of the UR had received a COVID-19 vaccine, which was offered to EU citizens as their first dose. However, some reported receiving the Chinese CoronaVac or Indian Covishield vaccine. As with the vaccines provided to EU citizens, the latter two vaccines are more effective at preventing severe COVID-19 outcomes than at preventing SARS-CoV-2 infection (27).

For UR, the key motivation for the next COVID-19 immunization was protecting one’s health (27.1% of responses). Protecting family health was rated second; however, only 13.3% of UR chose this reason, followed by protecting community health and co-workers’ health. Other factors, such as the possibility of travel, work or school requirements, return to social life, or government recommendations, did not play a significant role in their decisions. Keser et al. reported that such a pattern is typical among risk-averse, compliant individuals and accounts for their motivation to receive the COVID-19 vaccine to protect themselves and others from the virus (28). The same is true for female participants, who are particularly motivated by issues of protection. By contrast, as Cato et al. concluded, risk-tolerant and less altruistic individuals are less willing to be vaccinated (29).

### 4.3 Factors influencing vaccination

UR, who had been infected with SARS-CoV-2, were more than seven times more likely to be vaccinated. Consistent with our findings, some evidence indicates that prior SARS-CoV-2 infection was positively associated with COVID-19 vaccine uptake (30–38). For instance, a systematic review and meta-analysis of COVID-19 vaccination acceptance across global populations by Nindrea et al. revealed that exposure to COVID-19 was a significant factor (OR = 2.34) associated with vaccine acceptance (39). Several studies have examined the role of personal experience in the protection motivation model. According to some authors, it is the most potent stimulus of protective behavior (40–42). Experiencing a disease is likely to increase concern in some conditions. This results in increased self-protective behavior (43).

Additionally, we found that prior influenza vaccination is associated with more than a threefold increase in the likelihood of COVID-19 vaccination uptake. This demonstrates the potential facilitative role of a reliable vaccination experience in building and enhancing UR’s confidence in being immunized. A systematic review and meta-analysis also confirmed this phenomenon (34, 44, 45). Adu et al. found that 10 studies reported higher intention to vaccinate against COVID-19 among participants with prior vaccination experience, including influenza vaccination, than among those with no or minimal vaccination history (30). Notably, COVID-19 and seasonal influenza are likely to co-circulate (46). Patients with concomitant influenza and COVID-19 may have worse outcomes than those with COVID-19 alone. Therefore, lowering the risk of coinfections in susceptible patients is essential.

In our study, living in a large city in Ukraine (population >250,000) nearly doubled the likelihood of receiving a COVID-19 vaccine. Previous studies have also revealed that (37) place of residence was a significant factor in determining COVID-19 acceptance and uptake. In a survey conducted by Marzo et al., urban residents were more likely to support COVID-19 vaccine uptake (47). Rural residents reported greater hesitancy regarding the COVID-19 vaccine than urban residents. These findings are similar to other studies conducted in Bangladesh and the Philippines (48). Higher levels of accessibility, standard of living, and education are associated with higher vaccine uptake among people living in urban areas. Moreover, urban residents have greater access to a broader range of vaccine information sources.

The desire to travel also significantly increased the likelihood of COVID-19 vaccination (almost twofold). Previous work on tourism during the COVID-19 pandemic has shown that interest in travel may encourage vaccination adoption despite doubts (49). Notably, the results from our previous qualitative study suggest that although some UR may be hesitant to receive vaccination, they still do so despite low confidence in achieving the expected outcome (10). In other words, the requirement for vaccination might encourage otherwise hesitant UR to become vaccinated. The findings warrant further research into how travel desire can be used to initiate the adoption of positive health behaviors among UR.

Surprisingly, except for urban residence, we found no other sociodemographic factors significantly associated with increased COVID-19 vaccine uptake. This result is supported by the findings of Crawshaw et al., who did a systematic review exploring drivers of vaccine uptake and socio-demographic determinants of under-vaccination among migrants in the EU. The authors did not identify a strong overall association with factors such as gender or age (50).

### 4.4 Limitations

Several limitations in our study should be considered. First, given the limited resources, this survey was conducted at a single refugee center, which limited the generalizability of our findings. However, the study was conducted over three consecutive months, and completing the questionnaires was supervised by trained research team members. This facilitated obtaining valid responses. Furthermore, URs were represented in almost all provincial administrative regions across Ukraine. Second, demographic and lifestyle factors were based on self-report, which introduces the possibility of common method bias. Third, the closed-ended questionnaire used in this survey yielded limited information beyond the response options. Additionally, we were unable to verify self-reported vaccination; however, in other independent studies, there was a high degree of concordance between self-reported vaccination and respondents’ actual vaccination status (51, 52). This provides indirect evidence that self-reported COVID-19 and influenza vaccination status is a good proxy of verified vaccination status. Future research is needed to validate the concordance between the self-reported and registry-based vaccination records.

## 5 Conclusion

In this study, COVID-19 vaccination rates among UR were low, and a substantial proportion of respondents were reluctant to receive the vaccine. The observed level of immunization may be one of the factors that sustain the COVID-19 pandemic in host countries.

The study identified key predictors of vaccine uptake, including prior SARS-CoV-2 infection, a history of influenza vaccination, residence in larger Ukrainian cities, and willingness to travel abroad. These findings indicate that personal experience, attitudes, and demographic factors play an important role in vaccination decisions and should be considered in strategies to improve vaccine acceptance in this vulnerable population. As COVID-19 vaccines become more widely available, vulnerable migrant populations and interventions should not be neglected in research. Such effective, evidence-based interventions remain essential, especially given the need for booster doses of COVID-19 vaccines, the emergence of immune-escape variants, and the waning of population immunity (53–58).

## Data Availability

The original contributions presented in the study are included in the article/supplementary material, further inquiries can be directed to the corresponding author.
